# Etiology of acute respiratory disease in fattening pigs in Finland

**DOI:** 10.1186/s40813-017-0065-2

**Published:** 2017-08-23

**Authors:** Minna Haimi-Hakala, Outi Hälli, Tapio Laurila, Mirja Raunio-Saarnisto, Tiina Nokireki, Taina Laine, Suvi Nykäsenoja, Kirsti Pelkola, Joaquim Segales, Marina Sibila, Claudio Oliviero, Olli Peltoniemi, Sinikka Pelkonen, Mari Heinonen

**Affiliations:** 1 Department of Production Animal Medicine, University of Helsinki, Paroninkuja 20, 04920 Saarentaus, Finland; 2 Finnish Food Safety Authority Evira, PO Box 198, 60101 Seinäjoki, Finland; 3 Finnish Food Safety Authority Evira, Mustialankatu 3, 00790 Helsinki, Finland; 4 Centre de Recerca en Sanitat Animal (CReSA, IRTA-UAB), Campus de la Universitat Autònoma de Barcelona, 08193 Bellaterra, Spain; 5 Departament de Sanitat i Anatomia Animals, Facultat de Veterinària, UAB, 08193 Bellaterra, Barcelona, Spain

**Keywords:** Pig, Respiratory, Pathogen, Actinobacillosis, Swine influenza, Ascaris, Necropsy, Acute, Outbreak

## Abstract

**Background:**

The objective of our study was to clinically and etiologically investigate acute outbreaks of respiratory disease in Finland. Our study also aimed to evaluate the clinical use of various methods in diagnosing respiratory infections under field conditions and to describe the antimicrobial resistance profile of the main bacterial pathogen(s) found during the study.

**Methods:**

A total of 20 case herds having finishing pigs showing acute respiratory symptoms and eight control herds showing no clinical signs suggesting of respiratory problems were enrolled in the study. Researchers visited each herd twice, examining and bleeding 20 pigs per herd. In addition, nasal swab samples were taken from 20 pigs and three pigs per case herd were necropsied during the first visit. Serology was used to detect *Actinobacillus pleuropneumoniae* (APP), swine influenza virus (SIV), porcine reproductive and respiratory syndrome virus (PRRSV), porcine respiratory coronavirus (PRCV) and *Mycoplasma hyopneumoniae* antibodies. Polymerase chain reaction (PCR) was used to investigate the presence of porcine circovirus type 2 (PCV2) in serum and SIV in the nasal and lung samples. Pathology and bacteriology, including antimicrobial resistance determination, were performed on lung samples obtained from the field necropsies.

**Results:**

According to the pathology and bacteriology of the lung samples, APP and *Ascaris suum* were the main causes of respiratory outbreaks in 14 and three herds respectively, while the clinical signs in three other herds had a miscellaneous etiology. SIV, APP and PCV2 caused concurrent infections in certain herds but they were detected serologically or with PCR also in control herds, suggesting possible subclinical infections. APP was isolated from 16 (80%) case herds. Marked resistance was observed against tetracycline for APP, some resistance was detected against trimethoprim/sulfamethoxazole, ampicillin and penicillin, and no resistance against florfenicol, enrofloxacin, tulathromycin or tiamulin was found. Serology, even from paired serum samples, gave inconclusive results for acute APP infection diagnosis.

**Conclusions:**

APP was the most common cause for acute respiratory outbreaks in our study. SIV, *A. suum*, PCV2 and certain opportunistic bacteria were also detected during the outbreaks; however, viral pathogens appeared less important than bacteria. Necropsies supplemented with microbiology were the most efficient diagnostic methods in characterizing the studied outbreaks.

## Background

Porcine respiratory disease complex (PRDC) is a syndrome caused by mixed viral and bacterial pathogens together with environmental, managerial and genetic factors [1]. A combination of pathogens are involved, e.g. viruses such as porcine reproductive and respiratory syndrome virus (PRRSV), porcine circovirus type 2 (PCV2), swine influenza virus (SIV), porcine respiratory coronavirus (PRCV), and various bacteria e.g. *Actinobacillus pleuropneumoniae* (APP), *Mycoplasma hyopneumoniae* (MHyo)*, Mycoplasma hyorhinis, Haemophilus parasuis (H.parasuis), Pasteurella multocida* and *Streptococcus suis* [1–6]. Often it remains unclear which the primary pathogen is and which one is acting as a predisposing agent for other infections or as a secondary infection [2, 6, 7]. Many of these pathogens can also be found in clinically healthy pigs, but they are detected more often in pigs with respiratory symptoms [8]. The pathogenesis of multifactorial PRDC is difficult to determine, because primary and opportunistic pathogens modify their impacts in different cases [2].

Pathogens involved in PRDC vary considerably in various countries, regions and herds over time. In Finland, the prevalence of porcine respiratory pathogens differs substantially from the situation in continental Europe. The country has been free of ADV, PRCV and PRRSV for decades. Also, Finland is nearly free of swine enzootic pneumonia. In 2015, MHyo was detected in only one Finnish pig herd [9], and most Finnish pig production (97%) is included in the national health programme [10] requiring the absence of this pathogen. On the contrary, APP is a common pathogen causing respiratory problems [9]. SIV is a newcomer in the country. Avian-like H1N1 swine influenza A virus was found in the Finnish pig population for the first time in 2008 and A(H1N1)pdm09 influenza virus in 2009 [11]. PCV2 is a pathogen commonly found in the Finnish pig population, and many herd owners control it by vaccination. However, its role in respiratory infections in Finland has not been studied earlier. Overall, assessing which respiratory pathogens are involved in acute respiratory disease outbreaks in a country lacking major viral pathogens is very important.

The objective of our study was to clinically and etiologically investigate acute outbreaks of respiratory disease in Finland. This field study also aimed to evaluate the clinical use of various methods in diagnosing the respiratory infections under field conditions and to describe the antimicrobial resistance profile of the main bacterial pathogen(s) found during our study.

## Methods

### Study population

This case-control study was carried out between May 2011 and January 2014 in finishing or farrow-to-finish pig herds in southern and southwestern Finland. Practicing herd veterinarians and herd owners in the area were asked to contact the research group when acute respiratory symptoms became apparent in finishing pigs. Veterinarians were informed of the study via several emails, letters and at veterinary meetings along with an announcement in the Finnish Veterinary Journal. Herd owners were informed at farmer meetings organized by the major slaughterhouses. Case herds were included in the study if they had at least one pig room with finishing pigs displaying a cough, fever and lowered appetite. A total of 20 case herds were enrolled in our study.

Control herds (*N* = 8) were selected from herds taking part in another study during the same time period. They were selected as case herds according to similar geographical location, herd size and type (fattening and/or farrow-to-finish). The control herds consisted of herds where local practicing veterinarians regularly clinically checked for signs of disease, at least every 3 months, and no acute respiratory signs were diagnosed in the finishers at the time of our study.

Exact data on vaccination schemes were not available regarding case or control herds.

### Herd visits, clinical examination and sample taking

Researchers visited all the study herds on two occasions. The first visit to each case herd occurred within 3 days of farmers informing the research group about the respiratory symptom visible in their finishing pigs. On average, the visits occurred eight (standard deviation [SD] 6) days after the owner first observed the clinical signs and 26 (SD 13) days after the pigs had arrived to the fattening room. The second visit to case herds occurred 33 (SD 5) days after the first visit. The first visit to the control herds occurred 22 (SD 21) days after the feeder pigs arrived at the finishing rooms and the second visit was conducted 58 (SD 16) days later.

If a case herd had more than one room designated for finishers, the room exhibiting the most severe respiratory symptoms was selected for sampling. With the control herds, the room housing feeder pigs that had arrived 1–3 weeks earlier was selected. At the beginning of the first herd visit, each pig in the room was forced to stand up while researchers counted the number of coughing and sneezing episodes in the room for 5 min. A coughing/sneezing episode was defined as a single cough/sneeze or a set of continuous coughing/sneezing by a single pig. For the incidence ratio calculations, the number of sneezing and coughing episodes was related to the number of pigs in the room (number of episodes per 100 pigs for 5 min). In the case herds, farmers were also instructed to mark pigs displaying respiratory symptoms with colour markings before the herd visit. These pigs along with the ones observed coughing during the herd visit were selected for sampling. In both the case and control herds, 20 pigs were caught with a snout snare, their rectal temperature was measured and they were ear-tagged and blood-sampled for serological investigations of APP, SIV, MHyo, PRRSV and PRCV. In addition, nasal swabs were taken from 20 pigs in the case herds for SIV determination by PCR. Control herd pigs were clinically free from respiratory symptoms. They were examined for acute SIV infection only serologically, with paired serum samples, as SIV is known to be only found in nasal discharge during the acute phase of infection [12]. We estimated that with a prevalence of 15–20% and 15 samples, we would find at least one affected animal with 15 samples (95% confidence level). Similarly, with a prevalence of 20–25%, we would need 10 samples to find at least one affected animal. Based on these estimations, 15 paired serum samples were used for APP and SIV serology, 15 samples collected during first herd visit for PCV2 PCR analysis and 12 samples collected during second herd visit for PRRS and PRCV serology and 10 samples for MHyo serology.

In the case herds, three non-medicated pigs (13–14 weeks of age on average) with the most evident respiratory signs were euthanized and necropsied during the first herd visit. A lung and the heart of these pigs were sent to a laboratory for pathological, virological and bacteriological analyses, including antimicrobial susceptibility testing. No exact records of past usage of antimicrobial medication of case herds were available. No control herd pigs were available for necropsy.

The second visit to each herd included an interview with the owner, a count of the coughing and sneezing episodes of the pigs in the same room as during the first herd visit, and rectal temperature measurement and blood sampling of the ear-tagged pigs.

### Transport of samples to laboratory

The nasal swabs were inserted in a transporting media (Copan Universal Transport Medium, UTM-R, Copan Diagnostics Inc., Murrieta, USA) and surrounded with chilling gel packs before transportation to a laboratory, where they were analysed the next day. All blood samples were cooled to 4 °C and centrifuged at 3000 rpm for 10 min within 24 h after sampling. The sera were stored frozen in −18 °C until analysis. The lungs and hearts of the euthanized pigs were chilled with icepacks, sent to a laboratory and examined the next day. PCV2 analyses were performed in the laboratory of CReSA, Spain. All other analyses were conducted in the laboratory of the Finnish Food Safety Authority (Evira).

### Pathology

A total of 60 lung samples from euthanized pigs from 20 case herds were examined. Gross lesions in the lungs including consolidation, abscesses and fibrinous or fibrotic pleurisy and other findings were recorded. Tissue samples were taken from the affected areas in the lungs. The samples were fixed by immersion in 10% neutral formalin, embedded in paraffin, cut in 4-μm thick sections and stained with haematoxylin and eosin. Lung tissue samples were additionally taken, especially from pneumonic lesions, were submitted to bacteriological examination and a sample of the lung tissue was also submitted to be PCR-tested for SIV. These macroscopic lung lesions together with histological and bacteriological results were used to classify the herds.

### Bacteriology

For aerobic pathogen detection, the lung tissue samples were cultivated on bovine blood agar and incubated at 37 °C. In addition, for possible APP biotype 1 and *Haemophilus parasuis* isolation, the samples were cultivated on bovine blood agar with a *Staphylococcus aureus* streak and incubated under a 5% CO_2_ atmosphere at 37 °C. The small colonies showing enhanced growth around the *S. aureus* streak were isolated and confirmed by a positive Camp reaction. They were tested using multiplex PCR, which identified the species and APP serotypes 2, 5 and 6 [13]. The non-haemolytic NAD-dependent isolates with a negative CAMP reaction were further tested for *Haemophilus parasuis* using biochemical tests (oxidase, catalase, urease, fermentation of xylose, mannitol, inulin, trehalose and xylose supplemented with NAD and horse serum). All APP isolates obtained were tested for antimicrobial susceptibility.

The antimicrobial susceptibility of the APP isolates was determined using a broth microdilution method (penicillin, ampicillin, tetracycline, enrofloxacin, trimethoprim/sulfamethoxazole; VetMICTM, National Veterinary Institute SVA, Uppsala, Sweden) and a disk diffusion method (tiamulin 30 μg, tulathromycin 30 μg; Mast Diagnostics, Merseyside, UK) following the Clinical and Laboratory Standards Institute (CLSI) guidelines [14]. Susceptibility results were categorized as susceptible (S), intermediate (I) or resistant (R) using specific breakpoints for APP according to CLSI [15]. As no specific breakpoints have been determined for penicillin and trimethoprim/sulfamethoxazole, the interpretative criteria for the HACEK group were applied [16]. If the inhibition zone from the tulathromycin test showed a non-susceptible phenotype, susceptibility was further tested using the broth microdilution method (Sensititre®, Trek Diagnostics, East Grinstead, UK). APP ATCC 27090 was used as a quality control strain. If at least one APP strain was categorized as an I or an R in the antimicrobial susceptibility test, the entire herd was classified as either intermediate or resistant.

### Virology

#### Swine influenza virus

SIV RNA was extracted from the lung (*N* = 60) and nasal swab (*N* = 400) suspensions using a QiaAmp ViralRNA Mini Kit (Qiagen, Hilden, Germany). All samples used for virus detection were subjected to influenza A M-gene-specific real-time RT-PCR [17] and A(H1N1) pdm09 -specific real-time RT-PCR [18]. Results were expressed as detected or not detected.

#### Porcine circovirus type 2

PCV2 was tested by PCR on serum samples of the first 15 pigs out of the 20 collected in the case and control herds during the first farm visit. DNA was extracted from 200 μL of serum using BioSprint® 96 DNA Blood kit (Qiagen, GmbH, D-40724 Hilden) on the Bio Sprint 96 system (Qiagen). Positive and negative extraction controls were added to each extraction plate. DNA samples were processed by means of a standard PCV2 PCR method [19]. Negative controls were added after every 10 samples in each PCR plate. PCR products were run by electrophoresis on a 2% agarose gel stained with ethidium bromide. Results were expressed as the percentage of PCR-positive samples per herd.

### Herd classification based on microbiological and pathological outcome

The case herds were classified according to the pathology, bacteriology and virology of the three lung samples examined from each herd and the nasal swab samples examined for SIV.

A herd was considered to suffer from acute respiratory disease caused by APP (coded as CL-APP) when pigs examined pathologically exhibited either 1) typical pathological gross lesions for APP (various sizes of consolidated dark or grayish well-demarcated pneumonic areas or a consolidation with necrotic areas often together with local pleurisy) in at least two out of the three lung samples together with isolation of APP bacteria in two or three lung samples, or 2) characteristic pathological lesions in at least one lung sample and either a necrotic area indicative of APP infection or mild consolidated lesions in another lung sample combined with isolation of APP bacteria in all three lung samples.

A herd was considered to suffer from respiratory disease caused by an acute *Ascaris suum* (coded as CL-ASC) infestation when the following two criteria were fulfilled: 1) at least two out of the three pigs examined pathologically in these herds had compatible gross lesions (typically heavy, wet and mottled red lungs) compatible with an *A. suum* infestation, and 2) detection of gross ascarid larvae in the tracheal froth and/or in the histological sections of these lung samples. The lung samples defined as having lesions caused by an *A. suum* infestation had no other gross lesions characteristic of another specific respiratory pathogen.

A case herd was considered to suffer from acute swine influenza infection (coded as CL-SIV) if SIV was detected by PCR in at least one of the three examined lungs or in at least one of the 20 nasal swab samples.

Miscellaneous etiology (coded as CL-MISC) was considered if variable pneumonic lesions in the lung samples were observed and the abovementioned criteria were not fulfilled.

### Serology

#### Actinobacillus pleuropneumoniae

In the laboratory, we analysed samples of the first 15 pigs out of the 20 collected with paired samples available from both herd visits. APP antibodies were measured using two commercial test kits: IDEXX APP-ApxIV ELISA (IDEXX, Liebefeld-Bern, Swizerland) to detect antibodies against ApxIV toxin, which is produced by all known APP serotypes (19), and IDvet ID Screen APP 2 indirect ELISA (IDvet, Grabels, France) to detect antibodies against lipopolysaccharides (LPS) specific to APP serotype 2 (APP2) with a sensitivity of 82.9% and a specificity of 99.6% for IDEXX APP ApxIV Elisa and a specificity of 99.68% for IDVet APP2 Elisa. Both tests were performed according to the manufacturer’s instructions. The absorbance values (percentage of positive control) from the paired samples were compared. Seroconversion at the individual pig level was defined as an increase in absorbance. A herd was considered to suffer from an acute APP infection if at least one individual pig seroconverted based on at least one serologic test (ApxIV toxin or APP2 LPS) between the herd visits.

#### Swine influenza

The samples of the first 15 pigs out of the 20 collected with paired serum samples available from both herd visits available were analysed from each herd. All blood samples were tested with influenza A antibody ELISA (ID Screen® Influenza A Antibody Competition, IdVet, Grabels, France) according to manufacturer instructions. A sample was considered unclear when the competition percentage (S/N%) was 46–49% and positive when the competition percentage was ≤45%. If a herd had at least one unclear or positive blood sample (pig) in the ELISA test in either of the samplings (first or second), blood samples of that herd were further analysed using a hemagglutination inhibition (HI) test according to European Surveillance Network for Influenza in Pigs [20]. This was done with the antigens H1N1 (SW/Best/96), H1N2 (SW/Gent/7625/99) and H3N2 (SW/St. Oedenrode/96). All antigens were provided by GD Animal Health Service (Deventer, NL). A sample was considered HI positive if the HI titer was ≥1:20. Seroconversion at the individual pig level was defined as an increase in the HI titer between the first and second samples. A herd was considered to have an acute SIV infection if at least one individual pig seroconverted based on the HI test between herd visits.

#### Mycoplasma hyopneumoniae

Ten serum samples collected from the case herds during the second herd visit were examined for antibodies against MHyo. The antibodies were detected using a blocking ELISA commercial kit (MHyo ELISA, Oxoid, Basingstoke, UK), following manufacturer’s instructions. Samples with an optical density value less than 50% of the OD buffer control were interpreted as positive. The sensitivity and specificity of the ELISA test was 100% and 98%, respectively.

#### Porcine reproductive and respiratory syndrome virus and porcine respiratory coronavirus

Serological screening for PRCV and PRRSV were carried out in 12 samples collected from each case herd during the second herd visit. A commercially available TGEV/PRCV ELISA test (SVANOVIR®, Boehringer Ingelheim Svanova, Uppsala, Sweden) with a sensitivity of 93% and specificity of 97% and PRRSV (PRRS Virus Antibody Test Kit, IDEXX Laboratories, Hoofddorp, NL) with a sensitivity of 98.8% and specificity of 99.9% were used according to the manufacturers’ instructions. The PRCV test result was interpreted as negative when the percent inhibition was <45 and PRRSV test result negative when the S/P ratio was <0.4. A farm should be considered positive for PRRSV or PRCV if one or more samples were judged positive.

### Statistical data analyses

All statistical data analyses were performed using Stata 14.0 (StataCorp LP, Texas, USA) and the statistical significance of *p*-value <0.05 was used.

Body temperature of the studied case and control herd animals was compared using unconditional linear regression. Prevalence of clinical signs (coughing and sneezing) during the first and second herd visits and in the case and control herds were modelled as count data (number of coughing/sneezing episodes per 5 min) using a Poisson or negative binomial model.

The percentages of PCR-positive samples in serum (PCV2) were compared between the case and control herds using the Wilcoxon rank sum test, because the proportion was not normally distributed.

The proportion of APP and SIV seroconverted animals were calculated for both tests in each herd and compared between the case and control herds using unconditional linear regression. Herd status (acute APP or SIV infection yes/no) based on the serological results was compared between the case and control herds using the chi square test.

## Results

### Farm characteristics and season

Out of the 20 case herds, 19 had fatteners only and one was a farrow-to-finish herd. Case herds had an average of 901 (SD 511) fatteners per herd and 234 (SD 101) pigs in each room. The control herds consisted of six fattening and two farrow-to-finish herds with an average of 1075 (SD 400) fattening pigs per herd and 265 (SD 167) pigs per room. In the case and control herds, the pigs originated from an average of 3.7 (SD 6.4) and 1.1 (SD 0.3) different piglet-producing herds, respectively. The season of the first herd visit was relatively similar in case and control herds: spring 20% of case vs. 0% of control herds, summer 20% vs 25%, autumn 25% vs. 50% and winter 35% vs. 25%. Most of the herd visits were done during the year 2012 (60% of case and 62% of control herds), because that was the time when farmers and practicing veterinarians were actively informed about the project.

### Clinical signs

Average rectal temperatures of the pigs during the first herd visit were 39.7 °C (SD 0.3, *N* = 448) and 39.4 °C (SD 0.3, *N* = 160) in the case and control herds, respectively (*p* = 0.01). Corresponding figures for the second herd visit were 39.3 °C (SD 0.1, *N* = 427) and 39.3 °C (SD 0.2, *N* = 155) (*p* = 0.3) for the case and control farms, respectively.

An average 4.0 (SD 3.8, *N* = 17, case herds) and 0.2 (SD 0.3, *N* = 8, control herds) coughing episodes were counted per 100 pigs during the first herd visit. The incidence rate ratio (IRR) for coughing episodes (case vs. control herds) was 16.5 (*p* < 0.01) during the first herd visit. By the second visit the coughing episodes in the case herds decreased to the same level as in the control herds: 0.6 (SD 0.8) for the case rooms and 0.4 (SD 0.5) for the control rooms. No difference in IRR was observed for the coughing episodes during the second herd visit (IRR 1.5, *p* = 0.5).

During the first herd visit, case pigs averaged 12.2 sneezing episodes per 100 pigs (SD 11.1) and the control pigs averaged 5.5 (SD 5.3). The IRR for sneezing episodes (case vs. control herds) was 1.9 (*p* = 0.1) during the first herd visit. By the second visit, the sneezing episode count in the case herds had decreased down to the same frequency as in the control herds: 5.5 (SD 4.4) in the case herds and 3.9 episodes per 100 pigs in the control herds (SD 3.3; *p* = 0.2).

### Classification of respiratory infection status in the herds

Fourteen (70%) out of 20 case herds were classified as having respiratory infection caused by APP (CL-APP) based on the pathological and bacteriological results. Three case herds were classified with a miscellaneous (CL-MISC) respiratory infection unassociated with a specific pathogen and three other herds (15%) were diagnosed to suffer from acute *A. suum* infections (CL-ASC) based on the examination of lung samples. Two of the latter herds also had APP seroconverted pigs, and one of these two herds had gross lesions caused by APP in one lung sample.

Bacterial pathogens were isolated from the pig lungs of 18 (90%) case herds. Of these, 12 (66%) herds had APP only (11 of these herds were classified as CL-APP herds and one as a CL-ASC herd). Four herds (22%) had APP together with another bacteria (three of these herds were classified as CL-APP and one as CL-MISC herds) and two (11%) only had other pathogens (all classified as CL-MISC herds). No specific bacteria were detected in the pig lungs from two farms (10%). Other encountered bacteria included *Escherichia coli, P. multocida, S. aureus, Actinomyces* spp*., Streptococcus dysgalactiae subs. Equisimilis, S. suis and Streptococcus* spp. and gram- and CAMP-negative and NAD-dependent rodbacterium*.* The PCR testing showed all APP cultures to be APP serotype 2.

A herd-level summary containing relevant pathological, virological and serological results is presented in Table 1.

**Table 1:** Summary of diagnostic results of 20 case herds exhibiting a respiratory outbreak and of eight control herds with no respiratory symptoms in finishing pigs

Herd	Herd status	Herd classification^a^	Bacteriology	APP (serology)^b^	SIV (serology)^b^	SIV (detection)^c^	PCV2 (PCR)^d^
1	case	CL-APP	APP, Actinomyces sp.	Yes	Yes	No	3 (20%)
2	case	CL-ASC	No specific bacteria	No	No	No	0
3	case	CL-ASC	APP	Yes	No	No	0
4	case	CL-APP	APP	Yes	No	No	0
5	case	CL-APP	APP	Yes	No	No	0
6	case	CL-APP	APP	Yes	No	No	0
7	case	CL-MISC	gram-, CAMP-, NAD dependent rod-bacterium; S. aureus	Yes	Yes	No	0
8	case	CL-APP	APP	Yes	No	No	2 (13%)
9	case	CL-APP	APP	Yes	No	No	0
10	case	CL-APP	APP	Yes	No	No	0
11	case	CL-APP	APP	Yes	No	No	13 (67%)
12	case	CL-ASC	No specific bacteria	Yes	No	No	4 (27%)
13	case	CL-APP	APP, Str. dysgalactiae subsp. equisimilis, E.coli	Yes	No	No	0
14	case	CL-MISC	Str. suis, Streptococcus sp.	Yes	No	No	9 (47%)
15	case	CL-APP	APP, P. multocida	Yes	No	No	0
16	case	CL-APP	APP	Yes	Yes	No	0
17	case	CL-APP	APP	Yes	No	No	0
18	case	CL-APP	APP	Yes	No	No	2 (13%)
19	case	CL-APP	APP	Yes	No	No	1 (7%)
20	case	CL-MISC	APP, P. multocida	Yes	No	No	0
21	control	NA	NA	No	No	NA	1 (7%)
22	control	NA	NA	Yes	No	NA	0
23	control	NA	NA	Yes	Yes	NA	0
24	control	NA	NA	No	No	NA	0
25	control	NA	NA	Yes	No	NA	0
26	control	NA	NA	Yes	No	NA	1 (7%)
27	control	NA	NA	Yes	No	NA	0
28	control	NA	NA	Yes	Yes	NA	0

*Abbreviations*: *CL-APP* acute APP infection, *CL-ASC* acute Ascaris suum infection, *CL-MISC* acute infection of miscellaneous etiology, *APP Actinobacillus pleuropneumoniaen*, *A. suum Ascaris suum*, *S. aureus Staphylococcus aureus*, *P. multocida Pasteurella multocida*, *E.coli Escherichia coli*, *Actinomyces sp. Actinomyces species*, *Haemophilus sp. Haemophilus species*, *Str. dysgalactiae subsp. equisimilis Streptococcus dysgalactiae subspecies equisimilis*, *Str. suis Streptococcus suis*, *SIV* swine influenza virus, *PCV2* porcine circovirus 2, *NA* not available

^a^Main necropsy diagnosis based on pathological and bacteriological results from three necropsied pigs

^b^Seroconversion in ≥1 out of 15 pigs sampled

^c^SIV detection with PCR from ≥1 nasal sample out of 20 pigs or from ≥1 lung sample of three necropsied pigs

^d^Number and percentage (in parentheses) of PCV2 positive samples per herd in PCR analysis

All lung and nasal swab samples were SIV negative. Thus, none of the case herds was diagnosed to suffer from acute SIV infection based on PCR even though some herds were serologically positive.

### APP antimicrobial susceptibility

Forty-four APP isolates obtained from 16 case herds were tested for antimicrobial resistance. APP isolates from six herds (38%) were intermediately resistant and one herd (6%) tested resistant to tetracycline. Isolates from one herd (6%) were intermediately resistant while two herds (13%) had isolates resistant to trimethoprim/sulfamethoxazole. Isolates from two herds (13%) were resistant to ampicillin and penicillin. Altogether, five isolates resistant or intermediately resistant to at least two different antimicrobials were detected from three herds. No resistance to florfenicol, enrofloxacin, tiamulin or tulathromycin was found. Minimum inhibitory concentration (MIC) distribution is presented in Table 2 and growth inhibition zone distribution of APP strains in Table 3.

**Table 2:** Minimum inhibitory concentrations (MIC) for six antimicrobial agents for the *Actinobacillus pleuropneumoniae* strains (*N* = 44) isolated from lung samples collected from 60 finishing pigs in 16 out of 20 case herds

	No. of isolates with MIC (μg/ml)												
Anti-microbial	≤0.015	0.03	0.06	0.12	0.25	0.5	1	2	4	8	16	32	%Res
PEN				8	16	17		1	2				7
AMP						41			3				7
TET						27	16				1		39
ENR			38	3	3								0
SXT^a^					38	2	2	2					9
FFN							44						0

MICs equal to or lower than the lowest concentration tested are given as the lowest concentration

*Abbreviations*: *PEN* penicillin, *AMP* ampicillin, *TET* tetracycline, *ENR* enrofloxacin, *SXT* trimethoprim/sulfamethoxazole, *FFN* florfenicol, *%Res* Percentage of intermediately resistant or resistant APP isolates out of all isolates

^a^concentration of trimethoprim given, in concentration ratio 1/19

**Table 3:** Growth inhibition zones for two antimicrobial agents for 44 Actinobacillus pleuropneumoniae strains isolated from lung samples collected from 60 finishing pigs in 16 out of 20 case herds

Antimicrobial	No. of isolates with GIZ (mm)																	%Res
	6	7	8	9	10	11	12	13	14	15	16	17	18	19	20	21	22	
TIA									9	10	15	4	1	2	2		1	0
TUL				1^a^	1	9	11	6	7	4	2	2		1				0

*Abbreviations*: *TIA* tiamulin, *TUL* tulathromycin, *GIZ* growth inhibition zone, *%Res* Percentage of intermediately resistant or resistant APP isolates out of all isolates

^a^The isolate was tested also with the broth microdilution method and was found susceptible to tulathromycin (MIC value 32 μg/ml)

### Serology

#### Actinobacillus pleuropneumoniae

Antibodies to ApxIV toxin were detected during the first sampling in 14 (70%) case and six (75%) control herds. Respectively, APP2 antibodies were found in six (30%) case and five (62.5%) control herds. Seroconversion to either ApxIV toxin or APP2 LPS was detected in at least one pig in 19 (95%) case herds and six (75%) control herds. These 19 case and six control herds were classified to suffer from an ongoing acute APP infection based on the serology results. No difference was observed in the occurrence of acute APP infection between the case and control herds (*p* = 0.1). Seroconversion to ApxIV toxin was detected in 68.8% (SD 26.7) of the individual animals in the case herds and in 67.5% (SD 43.7) in the control herds. Seroconversion to APP2 LPS was detected in 38.3% (SD 27.9) of individual animals in the case herds and in 38.9% (SD 27.8) in the control herds. No difference was found in the proportion of seroconverted animals between the case and control herds (ApxIV toxin *p* = 0.9, APP2 *p* = 0.9).

#### Swine influenza virus

During the first sampling, SIV antibodies were found in pigs in eight (40%) case and three (37.5%) control herds. Three out of 20 case (15%) and two out of eight control (25%) herds were classified with an ongoing acute SIV infection at the time of the herd visits based on the seroconversion of at least one sampled pig. No difference was found in the number of acute SIV herds between the case and control herds (*p* = 0.5). On average, 5.9% (SD 9.1) of individual animals had seroconverted in the case herds and 8.5% (SD 12.8) in the control herds based on the HI test. No difference was found in the proportion of seroconverted animals between the case and control herds (*p* = 0.7).

#### Other pathogens

All samples tested were negative against PRRSV, Mhyo and PRCV antibodies.

### Porcine circovirus type 2

Six out of 20 (30%) case herds and two out of eight (25%) control herds had at least one PCR-positive serum, respectively. A total of 29 PCV2 PCR-positive samples with mean percentage of positive samples per herd 9.7% (SD 18.2) were detected in the case herds and one positive (1.7%, SD 3.1) in each control herd. No difference was observed in the proportion of PCV2 PCR-positive samples between the case and control herds.

## Discussion

*Actinobacillus pleuropneumoniae* was the most common cause of acute respiratory outbreaks in the studied finishing pig herds. However, SIV, *Ascaris suum*, PCV2 and certain opportunistic bacteria appeared to cause concurrent infections, potentially contributing to the respiratory disease outcome. SIV and PCV2 were detected also in control herds suggesting possible subclinical infections in these herds. Serology alone was not effective in determining the cause of a respiratory outbreak, but pathology and bacteriology were considered useful in reaching a complete diagnosis. In addition to this, bacteriology together with antibiotic resistance determination was valuable in selecting the correct medication to be used, which is important in practice.

Clinical examination of the case herd pigs revealed respiratory signs including higher rectal temperature and coughing when compared to the control herd pigs. We were unfortunately unable to acquire exact data on the mortality in the rooms, because some animals in several herds were moved before or between herd visits to other rooms housing sick animals. However, the clinical signs in the herds were quite mild compared to those reported in experimental studies [21]. During the first herd visit, the average rectal temperature of the case pigs was 39.7 °C and by the second herd visit it was at the same level as in the control herds. In a study by Loeffen et al. [22], pigs in 15 respiratory outbreaks, caused by similar pathogens as in our study, showed respiratory symptoms with fever rising to 40–42 °C, which is much higher than the body temperature measured in our study. We cannot rule out missing the peak body temperature in our study pigs, as this might have happened earlier than our first herd visit. In addition to coughing, sneezing was very commonly heard in our study herds. However, sneezing was also diagnosed frequently in the control herds and its occurrence did not decrease by the second herd visit, indicating a persistent cause present in all herd types.

APP2 was the main causative agent for acute respiratory infections in Finnish finishing pigs. Previous Finnish studies screening the APP serotypes present in the country have revealed that APP2 is a common serotype together with several others [23, 24]. However, these older studies detected only antibody prevalence, but did not establish the connection between antibody prevalence and clinical disease. Our present study considered APP2 to be the main etiological agent of respiratory outbreak in the majority of herds (14 out of 20). In addition, APP2 was isolated from two other herds: one herd with miscellaneous etiology of its respiratory outbreak and one herd with lung lesions caused by *A. suum*.

It is difficult to compare the role of APP in respiratory infections in various countries, as study herds have usually not been selected and sampled in the same way as in our study. A study on clinical outbreaks carried out in the Netherlands in a manner similar to ours found APP to be the most likely cause in five out of 16 clinical outbreaks [22]. Other researchers have often used slaughterhouse data and/or serology, but pathological findings from samples taken during visible clinical symptoms have not been used. This is most likely due to the difficulty in carrying out such field studies. In France, a cross-sectional study on infectious agents in respiratory diseases was performed in 125 French swine herds without including any information concerning the clinical situation of the herds [25]. The researchers found that APP2 (serological diagnosis) was significantly associated with extensive pleuritis in the slaughterhouses, but not with pneumonia. In addition, an association between pneumonia or pleuritis in the slaughterhouses and seropositivity to APP was found in three other studies [26–28].

Serology has indeed been used in several studies examining APP causing respiratory infections in finishing pigs, but serological results have usually not been connected to clinical findings or acute outbreaks. Herds e.g. in Spain [27], Italy [28], Canada [29] and Belgium [30] have commonly been found positive for APP antibodies. In our study, antibodies against ApxIV toxin and APP2 LPS were found in both the case and control herds already during the first herd visit. Detectable antibody levels have been reported 1–3 weeks after experimental infection [31]. Pigs in our study may have been in contact with APP long enough to enable some of them to have seroconverted earlier than the first herd visit. We know that the herd owners waited for an average of 8 days before communicating about a respiratory outbreak, which is quite a long time. Estimating the role of subclinical infections is also difficult. Subclinical APP infection is known to potentially cause seroconversion [29]. A subclinical APP infection was possibly ongoing in the control herds during the time of our study and the presence of an infection went unnoticed by the herd owner and the research personnel. Our study found that the use of serology in APP detection, in either single sampling or paired samples, for the diagnosis of acute respiratory disease in field conditions is of little value, because no exact information of the initiation time of the infection is available and because of subclinical infections. However, when the beginning of an infection is known, as is often the case in experimental studies, or when the course of the infection is followed [32], serology remains a valuable diagnostic tool [30].

Three out of 20 outbreaks had a miscellaneous etiology. The pathology and bacteriology of these herds revealed findings incompatible with the set criteria for acute APP or SIV infections and the presence of bacteria such as APP, *E. coli, P. multocida, S. aureus, Actinomyces* spp*., S. dysgalactiae subs. Equisimilis, S. suis* and *Streptococcus* spp. APP serology showed seroconversion in each of these herds. SIV seroconversion additionally happened in one herd. Other researchers have also found that reaching a proper diagnosis is not always easy under field conditions despite using several diagnostic methods. Researchers studying respiratory outbreaks in 16 herds in the Netherlands could not reach a definitive diagnosis in four herds [23]. They concluded that secondary bacteria might have played a role in the clinical outbreaks where no evident cause could be found. It is also possible the primary pathogen could not be identified in our study, despite the utilization of several diagnostic methods.

*Ascaris suum* infection was found to be the main cause of respiratory clinical signs in three case herds (15%). At least in Finland this pathogen has been considered a minor disease agent, especially in modern management systems with concrete floors and without outdoor access. It is widely known that *A. suum* can cause verminous pneumonia, as the larvae migrate through the lung tissue during their lifecycle [33]. Also, slaughterhouse statistics from Finnish yearly figures of approximately 2 million slaughter pigs confirm the importance of *A. suum* infestations (Finnish Food Safety Authority, personal communication). Liver condemnations due to milk spots caused by *A. suum* were recorded in an average 6.5% of the finishing pigs slaughtered during the years 2010–2015. Other, more specific diagnostic methods for ascarids, have shown the prevalence of this parasite to be high. For example, antibodies against *A. suum* were observed in 39% of the study herds in a Danish study on finishing pigs [34]. A higher prevalence was found in Serbia when using the flotation method, with approximately 50% of swine herds being *A. suum* positive [35]. Ascariasis is a clinically relevant disease, as it can cause production losses [36] and impair the immunity achieved by vaccinations [37]. Also, proper diagnosis is of utmost importance. The administration of antimicrobial agents is useless as a treatment method against ascariasis.

Viral pathogens appeared to be less important as a cause of acute respiratory symptoms in finishing pigs in our study. The significance of SIV infection varies in other studies. For example, a clinical field study in the Netherlands showed SIV to be the most frequent main cause of a clinical outbreak in 16 herds [21]. In a recent study in Brazil, nearly 70% of the nasal swab samples taken from piglets expressing signs of respiratory disease were PCR-positive for SIV. Furthermore, SIV was the most common finding in the virological evaluations of diseased animals showing lung lesions [38]. However, studies also exist in which SIV is detected from pigs suffering from respiratory symptoms or slaughtered finishing pigs with lung lesions, but other pathogens are more frequently observed [6, 8, 39]. Typically for SIV, the virus is often detected in combination with other pathogens [6, 38]. In our present study, SIV was not found in the nasal swabs or lung samples and none of the case herds were therefore classified to suffer from an acute respiratory infection caused by SIV. However, results from the nasal swabs might be at least partly false-negative because our nasal swab sampling took place fairly late compared to optimal timing. Herds were visited approximately 8 days after clinical signs commenced and, therefore, it might be more correct to designate case herds as suffering from sub-acute respiratory disease instead of acute respiratory disease, especially in the case of SIV infection. Nasal swabs should be taken within 4 days after infection onset to attain the optimal detection of SIV [12]. Serology revealed that three case herds out of 20 appeared to have had a concurrent SIV infection. Two out of the eight control herds also had pigs that had seroconverted and possibly suffered from subclinical SIV infection. In addition, both the case and control herds had antibodies already during the first herd visit. SIV serology, similarly to APP serology, should be understood more as a monitoring tool rather than as a diagnostic one. Nowadays, a convenient diagnostic sample is oral fluid, since at a population level, the presence of a pathogen may be detected for a longer period [40].

PCV2 has been associated with several disease syndromes collectively named porcine circovirus diseases [41]. The role PCV2 plays in PRDC has been suggested to always involve interaction or synergism with other respiratory pathogens [42]. The proportion of PCV2-positive animals was similar in the case and control farms of our study. PCV2 is a ubiquitous virus and hence PCR-positive animals occurring on a farm is very likely irrespective of the farm’s disease status. When blood sample analysis is based only on a standard PCR method (positive and negative, but no quantification of viral counts) without histopathology or detection of the virus in lymphoid tissues, the method is not sufficient for establishing the actual role of PCV2 in clinical disease. However, we did not see compatible PCV2 gross pathology during the field autopsies carried out on the case farms. Based on the lack of typical circovirus gross pathology and no difference in the proportion of PCR-positive animals between the case and control farms, we concluded that PCV2 probably did not play a major role in acute respiratory disease detected on the case farms despite the pathogen being present on farms. Vaccinating pigs against PCV2 infections is a very common preventive measure in Finland, and this has most likely contributed to the low occurrence of the pathogen.

According to our hypothesis, certain pathogens causing acute respiratory symptoms in pigs, namely Mhyo, PRRSV and PRCV, were not found in the studied Finnish herds. Especially Mhyo and PRRSV are important pathogens involved in respiratory infections in many pig-producing countries despite PRRSV not being detected in certain countries e.g. Finland [6, 8, 24, 43]. Certain PRCV strains can also contribute to respiratory disease [44]. The lack of these pathogens as causative agents of respiratory outbreaks in Finland makes the situation of finishing pig herds quite different and favourable compared to respiratory disease scenarios in several countries located across the world. The absence of these pathogens may have a significant impact on the prevalence of other respiratory pathogens. However, the similar disease situation is present also in specific pathogen free herds in other countries than Finland, and these farms and their veterinarians might benefit from obtained results.

The vaccination history of study animals may have had some influence on serological results. We do not know the exact vaccination scheme utilized for study animals. However, we know that at the time of the study, Finnish sow herds generally vaccinated all sows against erysipelas, parvovirus and colibacillosis and a vaccination of piglets against PCV2 is very common in the country. We also know that very few (most likely none of the herds in our study) herds vaccinated against APP or SIV. Based on that estimation, it is unlikely that vaccinations would have had any significant effect on APP serology.

In our present study, APP strains were susceptible to most of the tested antimicrobials. Only tetracycline resistance was detected in more than 10% of the isolates. Similar results have been found in other European countries. Thus, resistance to tetracycline among porcine APP is a growing problem. Other studies have occasionally observed resistance to penicillin, ampicillin and trimethoprim/sulfamethoxazole [45–49]. The Ministry of Agriculture and Forestry in Finland issued its first national recommendation for prudent antibiotic use in animals as early as 1996 [50]. Currently, the first-choice antimicrobial agent recommended in APP infections is G-penicillin and the second choice is tiamulin or tetracycline. It is notable that a few isolates were found to be resistant also to penicillin, ampicillin and trimethoprim/sulfamethoxazole, which further emphasizes the need to investigate the resistance of APP strains when selecting the appropriate treatment.

From a practical standpoint, the field necropsies supplemented with microbiological analysis was the most valuable diagnostic tool combination for detecting the main cause of acute respiratory infections in our study. Field necropsies have several advantages: the technique is simple, inexpensive and not pathogen-specific, preliminary results are promptly available and antimicrobial susceptibility results can be obtained from bacteria isolated from lesions. However, field necropsies are disadvantageous, since if acute disease is not leading to mortality, euthanasia should be performed. In acute respiratory outbreaks, field necropsies, sample-taking and antimicrobial susceptibility testing are extremely important, because resistance to certain recommended antimicrobials does exist. Susceptibility testing is necessary not only from the field veterinarian’s and single pig herd’s point of view, but also from a national policymaker’s perspective. Serology cannot be used alone in diagnosis, but offers detailed information about possible pathogens causing mainly subclinical infections. Also other diagnostic methods could be used. In addition to the already mentioned oral fluids, tracheobronchial swabs [51] or lavage [52] would be of help. Their disadvantage is the need for special equipment and/or sedation of the pig, which might limit this sampling method under field conditions.

## Conclusions

APP serotype 2 was the most common cause for acute respiratory outbreaks in finishing pigs in Finland and *A. suum* or other opportunistic bacteria caused acute coughing episodes in some herds. Viral pathogens appeared to have a minor role in causing the clinical signs. Field necropsies supplemented with microbiological analysis were the most valuable diagnostic tool combination in detecting the main cause of the infections under field conditions. Bacterial isolation from the lungs was especially important in assessing antimicrobial susceptibility and for optimizing antimicrobial treatment, because some resistance, especially to tetracycline, was found among the APP strains causing disease. Serological diagnostics were not optimal in the diagnosis of the respiratory outbreaks of our study. Although several different diagnostic methods were used, the primary pathogen causing the outbreak remained questionable in some herds.
